# Early piperacillin exposure during continuous infusion in critically ill patients: the impact of renal and liver dysfunctions

**DOI:** 10.1016/j.aicoj.2026.100164

**Published:** 2026-09-21

**Authors:** Matthieu Conseil, Inès Lakbar, Alina Laurino, Suheda Evsen, Adeline Mathieu, Clément Monet, Fouad Belafia, Mathieu Capdevila, Jean-Joseph Bendjilali-Sabiani, Gérald Chanques, Audrey De Jong, Yoann Cazaubon, Samir Jaber

**Affiliations:** a Department of Anesthesia and Intensive Care unit, Regional University Hospital of Montpellier, St-Eloi Hospital, University of Montpellier, PhyMedExp, INSERM U1046, CNRS UMR, 9214, Montpellier, CEDEX 5, France; b Service of Pharmacology and Toxicology, University Hospital of Montpellier, Montpellier, France; c Pathogenesis and Control of Chronic and Emerging Infections (PCCEI), University of Montpellier, INSERM, Montpellier, France

**Keywords:** Piperacillin-Tazobactam, Underexposure, Overexposure, Therapeutic drug monitoring, Pharmacokinetics, Anti-bacterial agents, Critical care

## Abstract

**Objective:**

To assess early piperacillin exposure in critically ill patients receiving continuous infusion piperacillin-tazobactam, identify clinical determinants of underexposure and overexposure, and evaluate piperacillin concentrations across organ dysfunction phenotypes.

**Design:**

Retrospective, single-center observational study.

**Setting:**

A 20-bed medical-surgical intensive care unit of a university hospital.

**Patients:**

All consecutive adult critically ill patients receiving continuous-infusion piperacillin–tazobactam with at least one total plasma piperacillin concentration measurement.

**Interventions:**

None.

**Measurements and Main Results:**

Observed plasma piperacillin concentrations were measured at predefined time points (day 1, day 3 and day 7). Target attainment was defined as total plasma concentrations of 80–160 mg/L, corresponding to 4–8 times the minimum inhibitory concentration of *Pseudomonas aeruginosa*.

A total of 350 patients were included, contributing 775 piperacillin measurements. At first sampling (day 1), 30% of patients were underexposed, 35% achieved the target range, and 35% were overexposed. In multivariable analyses, underexposure was less frequent in older patients and in those with renal dysfunction or shock, while male sex was independently associated with a higher risk of underexposure. Overexposure was strongly associated with renal dysfunction, higher bilirubin concentrations, and higher daily piperacillin dose. Piperacillin concentrations were highest in patients with combined hepato-renal dysfunction.

**Conclusions:**

Only one-third of critically ill patients achieved target piperacillin exposure at initial therapeutic drug monitoring during continuous infusion. Early deviations from target concentrations were frequent and primarily determined by renal dysfunction despite routine dose adjustment. These findings support early individualized dosing strategies integrating organ function assessment and therapeutic drug monitoring to optimize piperacillin exposure.

## Background

Sepsis and septic shock remain among the leading causes of mortality in intensive care units (ICU) worldwide, despite advances in supportive care and antimicrobial therapy [[1], [2], [3], [4]]. Early administration of appropriate antibiotic treatment is a major determinant of survival in patients with sepsis or septic shock [5,6]. However, timely initiation alone is insufficient if adequate antibiotic exposure is not achieved.

In critically ill patients with sepsis, inadequate antibiotic exposure is frequent and clinically relevant. Insufficient exposure, or underexposure, has been associated with microbiological failure, persistent infection, and worse clinical outcomes [[7], [8], [9]]. Conversely excessive exposure, or overexposure, is increasingly recognised as harmful, being associated with drug-related toxicity, including neurological adverse events and kidney injury, even for β-lactam antibiotics traditionally considered safe [10,11].

Sepsis is characterised by profound and dynamic pathophysiological changes that substantially alter antibiotic pharmacokinetics. Increased volume of distribution related to capillary leak and fluid resuscitation, augmented renal clearance in the early phase of sepsis, and subsequent organ dysfunction during the course of illness all contribute to marked interindividual variability in antibiotic exposure [12]. As a result, fixed dosing regimens frequently fail to achieve predictable plasma concentrations in patients with sepsis or septic shock, even when guideline-recommended doses are used [7,9,13].

Piperacillin–tazobactam (PTZ) is one of the most commonly prescribed broad-spectrum β-lactam antibiotics in the ICU for the empirical and targeted treatment of sepsis [14]. Piperacillin is a hydrophilic antibiotic predominantly eliminated by the kidneys, and its antibacterial activity depends on maintaining sufficient drug concentrations over time relative to the minimum inhibitory concentration of the infecting pathogen [15]. Continuous infusion of PTZ has therefore been widely adopted to optimise pharmacodynamic target attainment in critically ill patients [15,16]. Nevertheless, substantial variability in plasma piperacillin concentrations persists despite continuous infusion, exposing patients to both underexposure and overexposure [13,17].

One of the major determinants of this variability in antibiotic exposure in sepsis is organ dysfunction [18]. Renal dysfunction directly alters piperacillin elimination, yet current evidence to guide β-lactam dose adjustment in critically ill patients with acute kidney injury remains limited and of low quality, with uncertainty regarding the timing and extent of dose reductions [19]. In addition, hepatic dysfunction and systemic inflammation may further modify pharmacokinetics through changes in protein binding and drug distribution [20]. When renal and hepatic dysfunction coexist, dosing adaptation may become even more challenging.

The primary objective of this study was to determine the proportion of critically ill patients with sepsis or septic shock achieving predefined target total plasma piperacillin concentrations during continuous infusion of PTZ and to identify risk factors associated with piperacillin underexposure and overexposure. The secondary objective was to evaluate piperacillin exposure according to organ dysfunction profiles, including isolated renal dysfunction, isolated hepatic dysfunction, combined hepato-renal dysfunction, and absence of organ dysfunction.

## Methods

### Study setting and population

We conducted a retrospective, observational, pragmatic pharmacokinetic study in a single center. During 2018 and 2020, all consecutive adult critically ill patients admitted to the ICU and treated with piperacillin-tazobactam were eligible for inclusion (piperacillin assays were unavailable in 2019). Exclusion criteria included pregnancy, age under 18 years and unavailable plasma piperacillin concentrations. The study was approved by the Institutional Review Board of Montpellier University Hospital (IRB-MTP_2020_03_202000405).

### Antimicrobial therapy

PTZ was administered as a continuous intravenous infusion initiated immediately after completion of a 4 g/0.5 g loading dose. Continuous infusion doses were determined by the treating physician according to local clinical practice, taking into account patient characteristics, including renal function and clinical severity. Piperacillin plasma concentrations were made available to clinicians, and therapeutic adjustments (including dose or infusion rate modifications) were performed at their discretion based on clinical judgment. According to local dosing practice during the study period, the continuous-infusion maintenance dose was selected at treatment initiation based on renal function and renal replacement therapy. Dose reduction could therefore be initiated on Day 0, before the first concentration measurement, in patients with severe renal impairment or receiving renal replacement therapy.

### Therapeutic monitoring of piperacillin

The day of piperacillin-tazobactam initiation (loading dose immediately followed by continuous infusion) was defined as Day 0. Total plasma piperacillin concentrations were subsequently measured at predefined time points (day 1, day 3, and day 7) using a validated ultra-high-performance liquid chromatography assay. Samples were taken 8−24 h after starting continuous infusion. These measurements reflect the combined effects of the loading dose and early continuous infusion. As piperacillin half-life may be prolonged in patients with severe renal impairment or receiving renal replacement therapy [21], steady state could not be assumed. All measurements, including subsequent samples, are therefore reported as observed plasma piperacillin concentrations.

Piperacillin TDM results were available approximately 24 h after sample collection. Analyses were performed on weekdays only, with no results available on weekends. Sampling at Day 1, Day 3, and Day 7 reflected routine clinical practice rather than a protocolized schedule and corresponded to key stages of patient management.

Total plasma piperacillin concentrations were quantified with a lower limit of quantification of 3 mg/L and validated linearity over a range of 3−300 mg/L; samples exceeding this range were analysed after dilution.

Target total plasma piperacillin concentrations were defined as 80−160 mg/L, in accordance with the French Society of Anaesthesia and Intensive Care (SFAR) guidelines [15]. Therefore, underexposure was defined as a total plasma piperacillin concentration <80 mg/L, target attainment as 80–160 mg/L, and overexposure as >160 mg/L.

The pharmacokinetic/pharmacodynamic target was defined as a free piperacillin concentration of 4× the MIC of the causative pathogen, with an upper limit of 160 mg/L (total concentration) to reduce toxicity [[22], [23], [24]].

As quantitative MIC values were not routinely available in our center (disk diffusion method), target attainment during empirical therapy was based on the EUCAST epidemiological cutoff value (ECOFF) for *Pseudomonas aeruginosa* (MIC = 16 mg/L) [25]. Given that microbiological results are typically available after approximately 72 h, early dose adjustments were guided by therapeutic drug monitoring and clinical parameters, including renal function. When microbiological results subsequently became available, they were used to guide further clinical management, including confirmation of antibiotic appropriateness, de-escalation, or additional adaptation of therapy.

### Data collection

Demographic, clinical, microbiological, and therapeutic data were collected, including disease severity scores: Sequential Organ Failure Assessment (SOFA) score [26] and the Model for End-Stage Liver Disease (MELD) score [27]. Renal function was evaluated at ICU admission and at each therapeutic drug monitoring (TDM) time point (Day 1, Day 3, and Day 7). Creatinine clearance was determined using 24-h urinary creatinine measurements, according to routine local practice during the study period.

### Outcomes

The primary outcome was target attainment at Day 1, defined as achievement of the predefined piperacillin concentration target (80–160 mg/L) at the first TDM measurement.

The secondary endpoint was piperacillin exposure across subgroups defined by organ dysfunction status (liver, kidney, combined liver-kidney dysfunction, augmented renal clearance and no dysfunction).

### Definition of organ dysfunction subgroups

Hepatic dysfunction was defined as a hepatic SOFA score >2 and/or cirrhosis, and renal dysfunction as a creatinine clearance <60 mL/min. Patients with both conditions were classified as hepato-renal, while those with neither were considered to have no organ dysfunction. Augmented renal clearance was defined as a measured creatinine clearance ≥130 mL/min in patients not receiving renal replacement therapy.

### Statistical analysis

Continuous variables are presented as mean ± SD or median [IQR], and categorical variables as number (percentage). Group comparisons used appropriate parametric or non-parametric tests, with a two-sided p-value ≤0.05 considered statistically significant.

Factors associated with piperacillin under- or overexposure were identified using multinomial logistic regression, with target concentration as the reference category. Results are presented as odds ratios (ORs) with 95% confidence intervals (CIs). Multivariable models were used to adjust for potential confounding factors.

Variables with p < 0.20 in univariable analyses were entered into multivariable models. Comorbid chronic conditions (cirrhosis and chronic kidney disease), as well as creatinine clearance and renal replacement therapy, were excluded from the multivariable models to avoid collinearity with organ dysfunction categories.

Measurements obtained from inadequately collected blood samples or from inaccurately documented drug administration were excluded to ensure data quality and reliability.

All statistical analyses were conducted using R software (version 4.4.3).

## Results

### Baseline patients’ characteristics

A total of 350 ICU patients were included, contributing to 775 piperacillin plasma samples obtained on Day 1, Day 3, and Day 7 following PTZ administration (Fig. 1). The overall population was predominantly male (238/350, 68%) with a mean age of 62.4 ± 15 years. Mean SAPS II was 46.9 ± 17 and mean SOFA score was 7 ± 4 upon admission. The mean BMI was 26.8 ± 7 kg/m², and the median creatinine clearance was 62.7 mL/min [26.9–129.7]. Renal replacement therapy (RRT) was ongoing in 28 patients (8%), 100 patients (29%) were in shock, and 124 patients (35%) required mechanical ventilation.

**Fig. 1:**
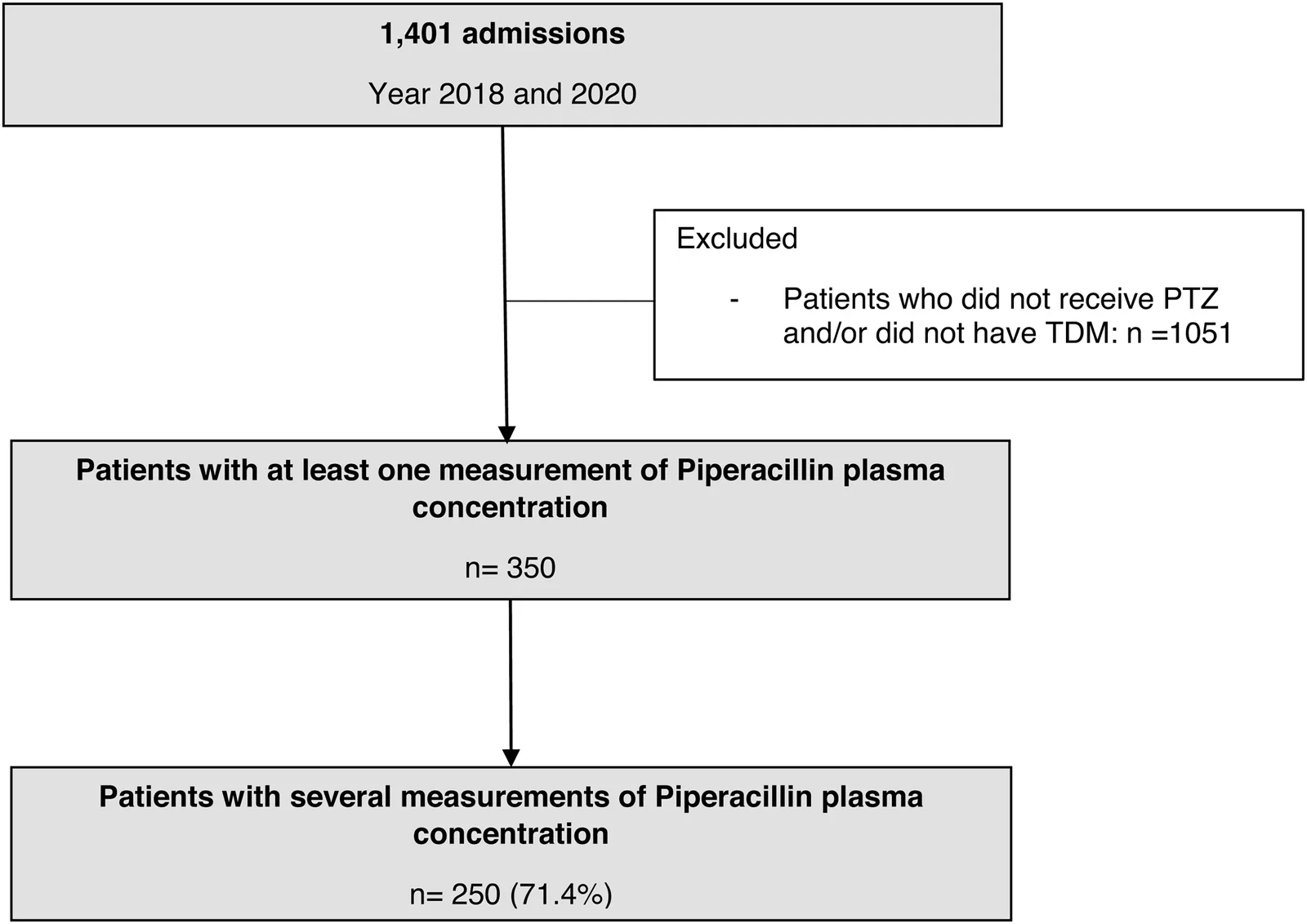
Flow-chart of the study. Abbreviations: PTZ: Piperacillin–tazobactam; TDM: therapeutic drug monitoring.

The main sources of sepsis were intra-abdominal for 58 patients (17%), pneumonia for 60 patients (17%) and bacteriemia for 43 patients (12.4%). Infection was documented in 238 patients (68%), among whom 137 (39% of the total cohort) had Gram-negative infections. Baseline characteristics according to exposure group are shown in Table 1.

**Table 1:** Baseline characteristics of the study population.

	Piperacillin concentration			Total
	Underexposure	Target	Overexposure	
	n = 104 (30%)	n = 123 (35%)	n = 123 (35%)	n = 350
*Characteristics at inclusion*				
Age (years), mean (±SD)	57.7 (±16)	64.3 (±14.2)	64.5 (±14.6)	62.4 (±15)
Gender male, number (%)	76 (73%)	79 (64%)	83 (67%)	238 (68%)
BMI (kg/m²), mean (±SD)	27.8 (±8.3)	26.5 (±6.6)	26.3 (±6.1)	26.8 (±7)
Weight (kg), mean (±SD)	81 (±24.9)	75.7 (±19.9)	75.9 (±19.5)	77.3 (±21.5)
Score				
SAPS II, mean (±SD)	43.1 (±15.9)	45.7 (±15.5)	51.3 (±18.5)	46.9 (±17)
SOFA, mean (±SD)	6 (±4)	7 (±4)	8.3 (±4)	7 (±4)
MELD, mean (±SD)	12 (±6)	15 (±7)	20 (±8)	16 (±8)
Comorbid conditions				
Cirrhosis, n (%)	22 (21%)	33 (27%)	42 (34%)	97 (28%)
Chronis kidney disease, n (%)	5 (5%)	22 (18%)	26 (21%)	53 (15%)
Surgical admission, n (%)	53 (51%)	55 (45%)	48 (39%)	156 (45%)
Category of infection, n (%)				
Pneumonia	21 (20%)	22 (18%)	17 (14%)	60 (17%)
Intra-abdominal	11 (11%)	19 (15%)	28 (23%)	58 (17%)
Bacteriemia	1 (1%)	7 (6%)	10 (8%)	43 (12.4%)
Others	4 (4%)	6 (4%)	5 (3%)	15 (4%)
Documented infection, n (%)	73 (70%)	82 (67%)	83 (67%)	238 (68%)
Gram-negative bacilli	49 (47%)	42 (34%)	46 (37%)	137 (39%)
*Characteristics on day of first Piperacillin assay*				
Creatinine clearance (ml/min), median [IQR]	129.2 [74.5–173.3]	70.8 [40.2–119.6]	25.5 [9.2–56.9]	62.7 [26.9–129.7]
Creatinine clearance categories, (ml/min), n (%)				
> 120	53 (51%)	28 (24%)	7 (6%)	88 (26%)
[60–120]	30 (29%)	34 (29%)	19 (16%)	83 (24%)
[30–59]	9 (9%)	27 (23%)	23 (19%)	59 (17%)
[10–29]	4 (4%)	14 (12%)	30 (25%)	48 (14%)
< 10 or RRT	8 (8%)	16 (13%)	42 (35%)	66 (19%)
RRT, n (%)	5 (5%)	12 (10%)	11 (9%)	28 (8%)
Shock, n (%)	18 (17%)	42 (34%)	40 (33%)	100 (29%)
Invasive ventilation, n (%)	39 (38%)	44 (36%)	41 (33%)	124 (35%)
Albumin (g/L), median [IQR]	30.0 [27.0–32.0]	28.0 [26.0–31.0]	29.0 [26.0–34.0]	29.0 [26.0–32.0]
Bilirubin (µmol/L), median [IQR]	15.0 [8.0–33.0]	25.0 [9.0–72.0]	28.0 [12.5–128.0]	22.0 [10.0–72.0]
Prothrombin rate (%), median [IQR]	76.0 [63.0–87.0]	72.0 [52.5–87.0]	65.0 [53.0–82.0]	71.0 [54.2–85.8]
*Piperacillin administration*				
Piperacillin dose per day (mg/kg), median [IQR]	184.3 [150.7–222.2]	183.9 [151.9–231.9]	183.9 [146.1–223.9]	183.9 [150.0–226.1]
Piperacillin concentration (mg/L), median [IQR]	58.9 [46.2–69.9]	115.6 [93.5–137.2]	233.5 [188.1–350.0]	120.2 [74.9–193.7]
*Outcomes*				
Duration of PTZ (days), median [IQR]	7.0 [5.0–11.0]	7.0 [4.0–11.0]	7.0 [4.5–12.0]	7.0 [5.0–11.0]
ICU mortality, n (%)	9 (9%)	13 (11%)	23 (19%)	45 (13%)

Abbreviations: BMI: body mass index; SAPS II: simplified acute physiology score II; SOFA: sequential organ failure assessment; RRT: renal replacement therapy, ICU: Intensive Care Unit; MELD, model for end-stage liver disease; IQR: interquartile range; SD; standard deviation; PTZ: piperacillin-tazobactam.

### Primary endpoint: piperacillin exposure

Among the 350 patients, at the time of the first piperacillin sampling, 30% (n = 104) were classified as underexposed (<80 mg/L), 35% (n = 123) were within the therapeutic target (80–160 mg/L), and 35% (n = 123) were overexposed (>160 mg/L) (Fig. 2). Across all 775 plasma piperacillin measurements, including initial and subsequent sampling, 31% (n = 240) were underexposed, 38% (n = 293) fell within the therapeutic target, and 31% (n = 242) were overexposed (Fig. S1).

**Fig. 2:**
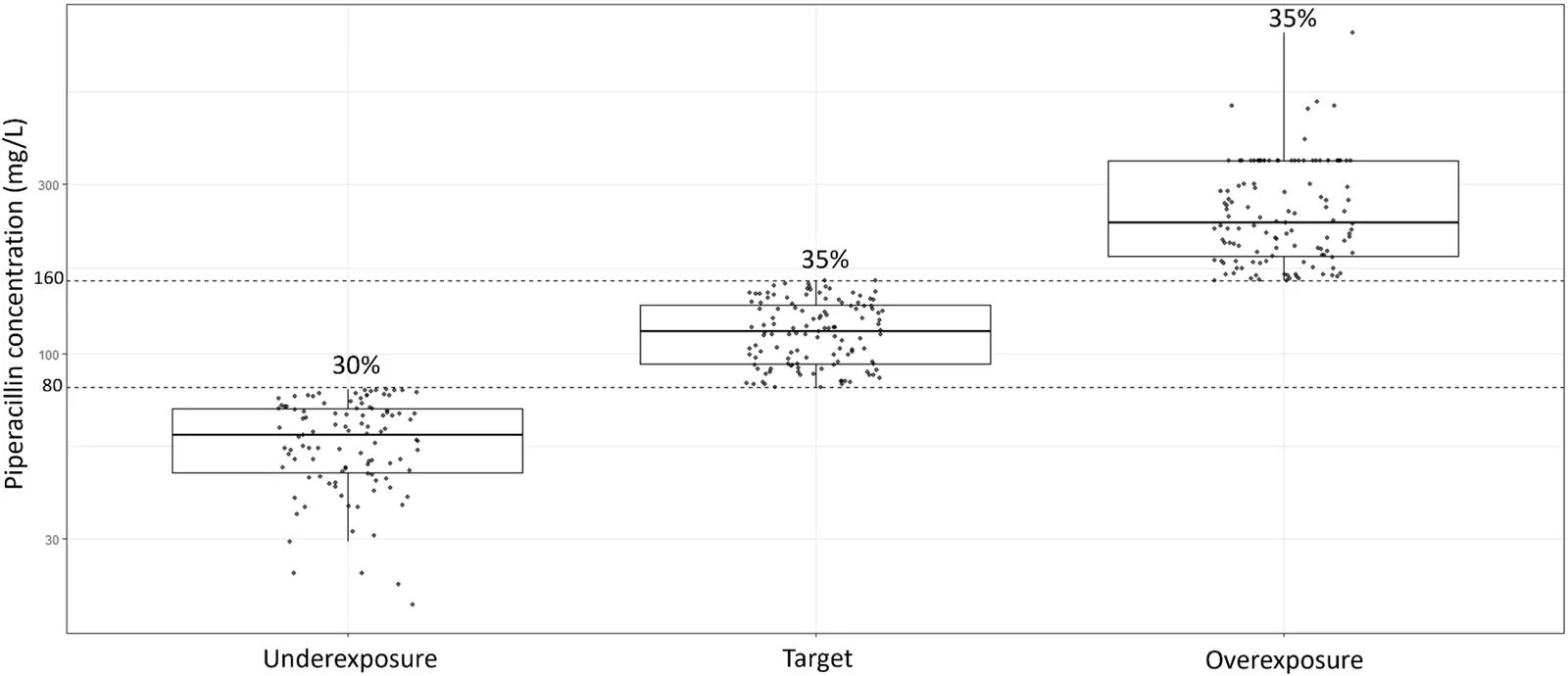
Observed plasma piperacillin concentrations by target category.

Median creatinine clearance was highest in the underdose group (129.2 mL/min [74.5–173.3]) and progressively lower in the target (70.8 mL/min [40.2–119.6]) and overexposed groups (25.5 mL/min [9.2–56.9]). Mean age increased across groups, from 57.7 ± 16 years in underdosed patients to 64.3 ± 14.2 years and 64.5 ± 14.6 years in the target and overexposed groups, respectively.

### Risk factors for underexposure

In univariate analysis, significant predictors of underexposure were as follows: age (OR 0.97 per year, 95% CI 0.96–0.99, p = 0.0017), low SOFA score (OR 0.93 per point, 95% CI 0.87–0.99, p = 0.033). Pre-existing chronic kidney disease markedly reduced the risk of underexposure (OR 0.23, 95% CI 0.08–0.64, p = 0.0046), as well as renal dysfunction (OR 0.35, 95% CI 0.19–0.66, p = 0.0013). In contrast, increased creatinine clearance was significantly associated with an elevated risk of underexposure (OR 1.01 per mL/min, 95% CI 1.01–1.01, p < 0.00001). The presence of shock was associated with lower odds of underexposure (OR 0.40, 95% CI 0.21–0.76, p = 0.0048), while invasive ventilation increased the likelihood of underdosing (OR 1.78, 95% CI 1.03–3.06, p = 0.037. High prothrombin activity was independently associated with underexposure (OR 1.01, 95% CI 1.00–1.03, p = 0.048) (Table 2).

**Table 2:** Multinomial logistic regression - underexposure versus overexposure.

	Underexposure vs Target		Overexposure vs Target	
	OR [95% CI]	P value	OR [95% CI]	P value
Characteristics at inclusion				
Age	0.97 (0.96–0.99)	**0.0017**	1.00 (0.98–1.02)	0.924
Gender male	1.51 (0.86–2.67)	0.155	1.16 (0.68–1.96)	0.591
BMI	1.03 (0.99–1.06)	0.184	1.00 (0.96–1.03)	0.848
Score				
SAPS II	0.99 (0.97–1.01)	0.230	1.02 (1.00–1.03)	**0.018**
SOFA	0.93 (0.87–0.99)	**0.033**	1.08 (1.01–1.15)	**0.018**
Comorbid conditions				
Cirrhosis	0.73 (0.39–1.36)	0.321	1.41 (0.82–2.44)	0.213
Chronis kidney disease	0.23 (0.08–0.64)	**0.0046**	1.23 (0.65–2.32)	0.52
Surgical admission	1.28 (0.76–2.17)	0.348	0.79 (0.48–1.31)	0.366
Liver dysfunction	0.60 (0.34–1.07)	0.081	1.55 (0.93–2.59)	0.093
Renal dysfunction	0.35 (0.19–0.66)	**0.0013**	4.17 (2.45–7.11)	**<0.000001**
Characteristics on day of first PIP assay				
Creatinine clearance (ml/min)	1.01 (1.01–1.01)	**<0.00001**	0.98 (0.98–0.99)	**<0.000001**
Shock	0.40 (0.21–0.76)	**0.0048**	0.93 (0.55–1.58)	0.787
Renal replacement therapy	0.47 (0.16–1.37)	0.166	0.91 (0.38–2.15)	0.827
Invasive ventilation	1.78 (1.03–3.06)	**0.037**	0.68 (0.41–1.12)	0.126
Albumin	1.03 (0.98–1.08)	0.263	1.05 (1.00–1.10)	**0.038**
Bilirubin (µmol/L)	1.00 (1.00–1.00)	0.389	1.00 (1.00–1.01)	**0.0125**
Prothrombin rate	1.01 (1.00–1.03)	**0.048**	0.99 (0.98–1.01)	0.295
Piperacillin administration				
Piperacillin dose per day	1.07 (0.98–1.16)	0.12	0.97 (0.90–1.05)	0.476

Abbreviations: BMI: body mass index; SAPS II: simplified acute physiology score II; SOFA: sequential organ failure assessment, CI: confidence interval, vs: versus.

In the multivariable model, older age was associated with a lower risk of piperacillin underexposure (OR 0.96 per year, 95% CI 0.94–0.98; p < 0.001), while male gender was associated with a higher risk (OR 2.60, 95% CI 1.34–5.21; p = 0.006). Renal dysfunction (OR 0.35, 95% CI 0.13–0.88; p = 0.030) and shock (OR 0.44, 95% CI 0.21–0.89; p = 0.024) were associated with reduced odds of underexposure. Prothrombin rate showed a borderline association (OR 1.02 per % increase, 95% CI 1.00–1.03; p = 0.064) (Table 3a).

**Table 3a:** Multivariate model of underexposure group versus target group.

	ß parameter	OR	95% CI	p-value
Age (per year)	−0.04	0.96	0.94 – 0.98	**<0.001**
Gender male	0.96	2.60	1.34 – 5.21	**0.006**
Renal dysfunction (Yes)	−1.06	0.35	0.13 – 0.88	**0.030**
Shock (Yes)	−0.82	0.44	0.21 – 0.89	**0.024**
Prothrombin rate (% per unit)	0.02	1.02	1.00 – 1.03	0.064

### Risk factors for overexposure

In the univariate analysis, the risk of overexposure was primarily associated with disease severity, reflected by both the SAPS II score (OR 1.02 per point, 95% CI 1.00–1.03; p = 0.018) and the SOFA score (OR 1.08 per point, 95% CI 1.01–1.15; p = 0.018), renal dysfunction (OR 4.17, 95% CI 2.45–7.11; p < 0.000001). Conversely, increased creatinine clearance significantly reduced the risk of overexposure (OR 0.98 per mL/min, 95% CI 0.98–0.99, p < 0.000001). Markers of hepatic impairment were also associated with overexposure, with higher serum albumin (OR 1.05 per g/L, 95% CI 1.00–1.10, p = 0.038) and elevated bilirubin concentrations (OR 1.00 per µmol/L, 95% CI 1.00–1.01, p = 0.0125) both associated with increased odds of overexposure (Table 2).

In the multivariable model based on the first sampling, renal dysfunction was the strongest independent predictor of piperacillin exposure (OR 7.07, 95% CI 3.44–15.25; p < 0.001). Higher bilirubin concentrations were also independently associated with overexposure (OR 1.00 per µmol/L, 95% CI 1.00–1.01; p = 0.025), as was a higher daily piperacillin dose (OR 1.15 per g/day, 95% CI 1.04–1.28; p = 0.010) (Table 3b).

**Table 3b:** Multivariate model of overexposure group versus target group.

	ß parameter	OR	95% CI	p-value
Renal dysfunction	1.96	7.07	3.44 – 15.25	**< 0.001**
Bilirubin (µmol/L)	0.004	1.00	1.00 – 1.01	**0.025**
Piperacillin dose (g/day)	0.14	1.15	1.04 – 1.28	**0.010**

Expanded analyses for factors associated with underexposure and overexposure including all dataset are reported in Supplementary Table S2.

### Secondary endpoint: piperacillin exposure according to organ dysfunction status

Patients were stratified into five groups according to organ function: augmented renal clearance (n = 84; 24.0%), isolated liver dysfunction (n = 41; 11.7%), isolated kidney dysfunction (n = 88; 25.1%), combined liver-kidney dysfunction (n = 59; 16.8%), and no organ dysfunction (n = 78; 22.3%) (Fig. 3). Median piperacillin daily doses were 16 g/day [16–16] in patients with augmented renal clearance, no organ dysfunction, and isolated liver dysfunction, 12 g/day [11–16] in patients with isolated kidney dysfunction and 12 g/day [12–16] in patients with combined liver-kidney dysfunction (Table S4). Despite these lower maintenance doses, patients with renal dysfunction and particularly those with combined hepato-renal dysfunction remained frequently overexposed, whereas patients with augmented renal clearance were predominantly underexposed (Fig. 3 and Fig. S3). Underexposure (<80 mg/L) occurred in 48/84 patients (57%) with augmented renal clearance, 24/78 (31%) with no organ dysfunction, 8/41 (20%) with isolated liver dysfunction, 8/88 (9%) with isolated kidney dysfunction, and 3/59 (5%) with combined liver-kidney dysfunction. In contrast, overexposure (>160 mg/L) occurred in 6/84 patients (7%) with augmented renal clearance, 19/78 (24%) with no organ dysfunction, 14/41 (34%) with isolated liver dysfunction, 52/88 (59%) with isolated kidney dysfunction, and 40/59 (68%) with combined liver-kidney dysfunction. A Sankey diagram illustrating individual transitions between exposure categories over time is provided in Fig. S4. It highlights frequent shifts between underexposure, target attainment, and overexposure, with limited persistence within a single category.

**Fig. 3:**
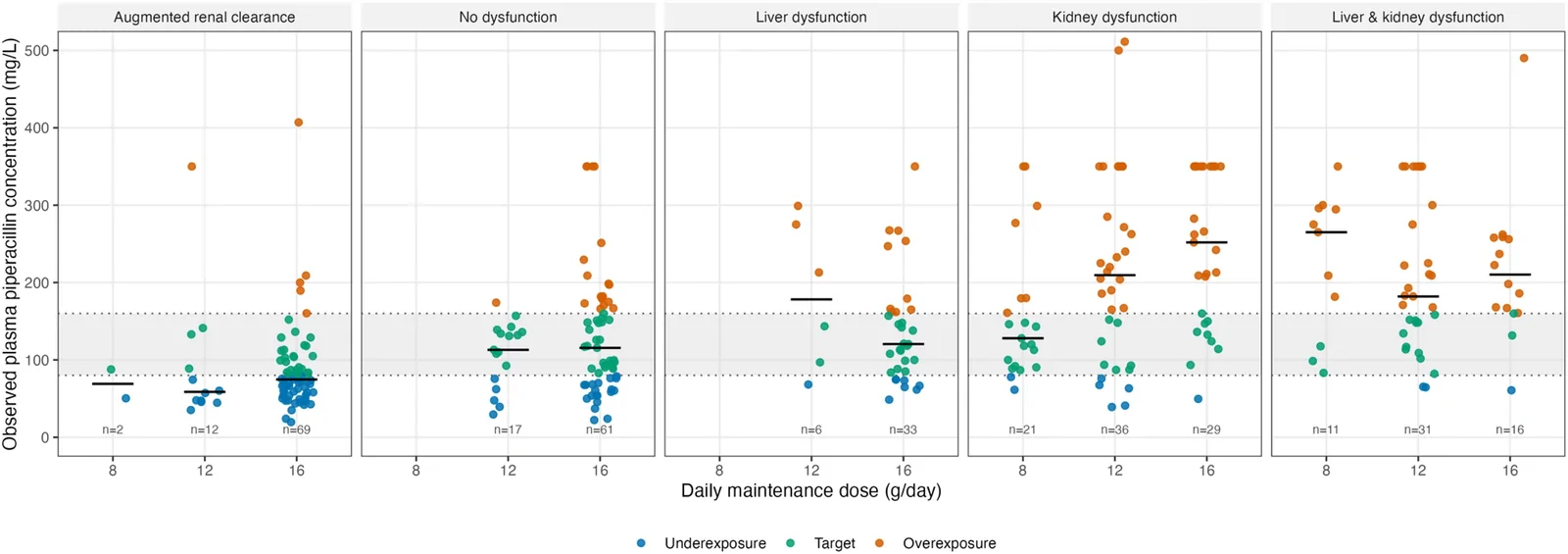
Observed plasma piperacillin concentrations according to daily maintenance dose and augmented renal clearance or organ dysfunction status at Day 1. Each point represents one patient. Colours indicate underexposure (<80 mg/L), target attainment (80–160 mg/L), and overexposure (>160 mg/L). The shaded area and dotted horizontal lines indicate the therapeutic target range. Black horizontal bars indicate the median concentration. Sample sizes are shown below each dose group. Analysis was restricted to the three principal maintenance doses (8, 12 and 16 g/day).

## Discussion

This study evaluated target attainment of continuous-infusion piperacillin-tazobactam in a large cohort of critically ill patients and investigated the influence of organ dysfunction on antibiotic exposure. We observed that target concentrations (80–160 mg/L) were achieved in approximately 35% of patients, whereas 30% were underexposed and 35% were overexposed at the time of the first piperacillin assay. Despite standardized dosing, piperacillin concentrations showed marked inter-individual variability, demonstrating that fixed dosing strategies are insufficient to achieve consistent pharmacokinetic targets in the ICU.

These findings are consistent with previous studies reporting high rates of target non-attainment with continuous infusion. Differences in the proportion of under- and overexposure across studies likely reflect variability in PK/PD targets and patient populations [28,29]. In line with prior work, we identified renal function as the primary determinant of exposure: preserved or augmented renal clearance was associated with underexposure, whereas renal dysfunction markedly increased the risk of overexposure. Augmented renal clearance (ARC), defined as creatinine clearance ≥130 mL/min, was observed in 84 (24%) patients in our cohort. In this subgroup, median piperacillin concentrations were lower (74.6 mg/L [IQR 54.4–100.2]) compared to the overall population, consistent with increased drug clearance. These findings support the known association between ARC and reduced β-lactam exposure and highlight the importance of considering this population in dosing strategies [[30], [31], [32], [33], [34], [35]]. Conversely, renal dysfunction was the strongest predictor of overexposure, confirming the central role of renal elimination in piperacillin pharmacokinetics. In addition, older age was consistently associated with a lower risk of underexposure; whereas male sex independently increased the risk of underdosing, aligning with prior studies [[36], [37], [38], [39]], and suggesting sex and age-related differences in pharmacokinetics beyond renal function alone.

The association between shock and reduced odds of piperacillin underexposure should not be interpreted as a protective effect of septic shock. Opposing pharmacokinetic mechanisms may contribute to piperacillin exposure in these patients. Reduced renal clearance may increase piperacillin concentrations, whereas capillary leak and fluid resuscitation may increase the volume of distribution of hydrophilic antimicrobials and thereby increase the risk of early underexposure [40]. In addition, according to local practice, maintenance doses could be reduced from Day 0 on the basis of renal function, before the first concentration measurement. The association observed in our study should therefore be interpreted in the context of this early dose adjustment and should not be used to support systematic early dose reduction in patients with septic shock.

In our study, piperacillin dosing differed across clinical groups, with lower median daily doses in patients with renal or combined hepatorenal dysfunction compared to those without, reflecting routine dose adjustment. However, this was not always sufficient to prevent drug accumulation. This likely reflects the dynamic nature of renal function in critical illness. Measured creatinine clearance and serum creatinine may not accurately capture real-time changes in drug elimination, particularly early in the ICU stay, as 24-h urine collections reflect prior renal function and creatinine changes may be delayed. Compared with shorter collection periods, the use of a 24-h urine collection may have introduced a temporal mismatch with the Day 1 piperacillin measurement and may have affected renal dysfunction or augmented renal clearance classification in patients with rapidly changing renal function.

While aggressive early β-lactam dosing may be appropriate in patients with preserved or augmented renal clearance, our data support the concept that initial maintenance dosing should be modulated according to functional elimination capacity in patients with acute kidney injury and in those with combined hepatic and renal dysfunction [41,42], rather than waiting for therapeutic drug monitoring to correct substantial early overexposure.

Notably, recent evidence has highlighted the limited and low-quality data available to guide β-lactam dose reductions in critically ill patients with acute kidney injury [19]. Our findings do not resolve this uncertainty but provide real-world exposure data suggesting that early overexposure is frequent in these patients under standard continuous-infusion regimens. Therefore, an integrated strategy combining (I) a loading dose to avoid early underexposure, (II) early adjustment of maintenance infusion based on renal function and organ dysfunction profile, and (III) rapid TDM within the first 24 h, may represent a pragmatic approach to balance efficacy and safety.

Although piperacillin is only moderately protein-bound (∼20–30%), hypoalbuminemia may increase the free fraction and modify piperacillin pharmacokinetics, particularly in the presence of renal dysfunction [20]. Hepatic cirrhosis has been associated with a ∼25% increase in piperacillin half-life, with prolonged elimination reported in patients with ascites [[43], [44], [45]]. In addition, in critically ill cirrhotic patients, excessive β-lactam concentrations have been observed in up to two thirds of cases [46,47]. Reduced prothrombin activity may therefore reflect the severity of hepatic dysfunction and act as a surrogate marker of cirrhosis-related alterations affecting drug disposition [15,48]. In our cohort, the exposure profile associated with isolated hepatic dysfunction was relatively close to that observed in patients without organ dysfunction, whereas overexposure was most frequent in patients with combined hepatic and renal dysfunction. This association may reflect the complex renal consequences of liver disease rather than reduced hepatic elimination of piperacillin. Cirrhosis may be associated with functional or structural kidney dysfunction, and liver failure may alter renal drug transporters involved in tubular secretion [49,50]. However, because we did not distinguish acute from chronic renal dysfunction or assess tubular secretory function, the underlying mechanism cannot be determined from our data. These findings support close therapeutic drug monitoring in patients with combined hepatic and renal dysfunction but do not establish a need for dose reduction based on hepatic dysfunction alone.

This study has limitations. Its retrospective design precluded standardized sampling times and protocolized dose adjustments following therapeutic drug monitoring. Residual confounding related to disease severity and unmeasured variables cannot be excluded. The use of a uniform target based on the ECOFF of *Pseudomonas aeruginosa* may have overestimated underexposure in infections caused by more susceptible organisms, for which lower piperacillin concentrations may have provided adequate pharmacodynamic exposure. In addition, administration-related factors may have influenced effective piperacillin delivery and measured plasma concentrations. Residual drug error, defined as the unintentional retention of medication within intravenous delivery systems, has recently been highlighted as a potential source of variability in antibiotic exposure in intensive care settings, particularly for continuous infusions [51]. Furthermore, we assessed total rather than unbound piperacillin concentrations, which may be particularly relevant in patients with altered protein binding, such as those with hypoalbuminemia or hyperbilirubinemia.

Nevertheless, the large sample size and the comprehensive assessment of organ dysfunction strengthen the robustness and clinical relevance of our findings.

## Conclusion

In critically ill patients receiving continuous-infusion piperacillin–tazobactam, only one-third achieved the recommended therapeutic target at initial drug monitoring. Piperacillin exposure was strongly determined by organ dysfunction profiles, with frequent and early deviations from target concentrations under standard dosing regimens. These findings suggest that early dosing strategies should account for functional elimination capacity, particularly in patients with acute kidney injury and combined hepato-renal dysfunction and support the use of early dosing adaptation and early therapeutic drug monitoring to optimize both efficacy and safety.

## Authors’ contributions

Data collection: SE, AM.

Study design: MC, ADJ, SJ.

Data analysis: AL, IL, CM, YC, JJB.

Data interpretation: AL, IL, MC, CM, ADJ, SJ.

Writing: AL, IL, SJ.

Overall manuscript review: AL, IL, MC, CM, GC, FB, MCa, ADJ, SJ, YC, JJB.

## Ethics approval and consent to participate

Not applicable.

## Consent for publication

Not applicable.

## Funding

None.

## Data availability

Available on reasonable request.

## Declaration of competing interest

SJ received consultant fees from Mindray, Fisher-Paykel, Baxter, Fresenius, Medtronic and Drager. AdJ received fees for presentations from Sanofi, Medtronic, Viatris. IL received fees from presentations Viatris and AOP Health.

No other potential conflict of interest relevant to this article was reported.
