# Supplementary material for: Early piperacillin exposure during continuous infusion in critically ill patients: the impact of renal and liver dysfunctions
Source: Ann Intensive Care. 2026 Sep 21;16:100164. doi: 10.1016/j.aicoj.2026.100164 (PMC13656968; doi:10.1016/j.aicoj.2026.100164)
Supplement: Supplementary file 1 [file mmc1.docx]

**SUPPLEMENTARY MATERIAL**

**Early piperacillin exposure during continuous infusion in critically ill patients: the impact of renal and liver dysfunctions**

Matthieu Conseil^1†^, Inès Lakbar^1†^, Alina Laurino^1^, Suheda Evsen^1^ , Adeline Mathieu^1^, Clément Monet^1^, Fouad Belafia^1^, Mathieu Capdevila^1^, Jean-Joseph Bendjilali^2,3^, Gérald Chanques^1^, Audrey De Jong^1^, Yoann Cazaubon^2,3^, Samir Jaber^1^

1. Department of Anesthesia and Intensive Care unit, Regional University Hospital of Montpellier, St-Eloi Hospital, University of Montpellier, PhyMedExp, INSERM U1046, CNRS UMR, 9214, Montpellier, CEDEX 5, France.

2. Service of Pharmacology and Toxicology, University Hospital of Montpellier, Montpellier, France.

3. Pathogenesis and Control of Chronic and Emerging Infections (PCCEI), University of Montpellier, INSERM, Montpellier, France

^†^Authors contributed equally

**Corresponding author:** Inès Lakbar, Département d’Anesthésie-Réanimation B (DAR B), 80 Avenue Augustin Fliche, 34295 Montpellier, France. Member of *Annals of Intensive Care* Editorial Board

Email: ines.lakbar@chu-montpellier.fr ; Tel: +33 467 33 72 71

**Ethics approval and consent to participate:** Not applicable

**Consent for publication:** Not applicable

**Availability of data and materials:** Available on reasonable request

**Competing interests:** SJ received consultant fees from Mindray, Fisher-Paykel, Baxter, Fresenius, Medtronic and Drager. AdJ received fees for presentations from Sanofi, Medtronic, Viatris. IL received fees from presentations Viatris and AOP Health.

No other potential conflict of interest relevant to this article was reported.

**Authors’ contributions:**

Data collection: SE, AM

Study design: MC, ADJ, SJ

Data analysis: AL, IL, CM, YC, JJB

Data interpretation: AL, IL, MC,CM, ADJ, SJ

Writing : AL, IL, SJ

Overall manuscript review: AL, IL, MC,CM, GC, FB, MCa, ADJ, SJ, YC, JJB

**Funding** None

**Keywords** Piperacillin-Tazobactam; Underexposure; Overexposure; Therapeutic Drug Monitoring; Pharmacokinetics; Anti-Bacterial Agents; Critical Care

**EXTENDED METHODS**

**Univariable multinomial logistic regression model (univariable analysis)**

In the first piperacillin concentration dataset (n = 350), piperacillin exposure was categorized according to total plasma concentration as underexposure (<80 mg/L), target exposure (80–160 mg/L), or overexposure (>160 mg/L), as defined in the main Methods. Exposure category was modelled as a three-level outcome, with the target exposure group used as the reference category.

Univariable associations between candidate predictors and exposure category were assessed using multinomial logistic regression (multinom function, nnet package). Each predictor was tested in a separate model (exposure category ~ predictor). For each model, analyses were restricted to observations with non-missing values for the predictor (complete-case per variable). Continuous variables (age, BMI, SAPS II, SOFA, creatinine clearance, albumin, bilirubin, prothrombin activity, and daily piperacillin dose) were entered as linear terms. Categorical predictors were prespecified and coded as follows: sex (Male vs Female [reference]); cirrhosis (Yes vs No [reference]); chronic kidney disease (Yes vs No [reference]); surgical admission (Surgical vs Medical [reference]); shock (Yes if shock vs No [reference]); invasive ventilation (Yes if intubation at admission or tracheotomy during stay vs No [reference]); renal replacement therapy (Yes vs No [reference]); liver dysfunction (Yes vs No [reference]); and renal dysfunction (Yes vs No [reference]).

Results are presented as odds ratios (ORs) with 95% confidence intervals (CIs) for Under vs Target and Over vs Target, obtained by exponentiation of model coefficients. Analyses were performed in R using the packages nnet and broom. All statistical analyses were conducted using R software (version 4.4.3).

**Multivariable logistic regression analyses (under- and overexposure)**

To identify factors independently associated with piperacillin underexposure and overexposure at the first sampling dataset, separate multivariable logistic regression models were constructed, each using target exposure (80–160 mg/L) as the reference category.

For the underexposure analysis, the dataset was restricted to patients with underexposed (<80 mg/L) or target-range concentrations. Piperacillin underexposure (<80 mg/L) was modelled as a binary outcome (underexposure vs target exposure) using a generalized linear model with a binomial link function. Candidate covariates were selected a priori based on clinical relevance and univariable associations, and included age, sex, SAPS II, SOFA score, bilirubin concentration, shock, invasive ventilation, daily piperacillin dose, surgical admission, liver dysfunction, and renal dysfunction.

For the overexposure analysis, the dataset was restricted to patients with overexposed (>160 mg/L) or target-range concentrations, and overexposure was modelled against target exposure using a similar binomial logistic regression framework. The final model included age, sex, BMI, SAPS II, SOFA score, renal dysfunction, liver dysfunction, bilirubin concentration, albumin concentration, daily piperacillin dose, invasive ventilation, shock, and surgical admission.

In both models, chronic kidney disease, cirrhosis, renal replacement therapy, and creatinine clearance were not included in order to avoid redundancy and collinearity with the predefined organ dysfunction variables. All continuous covariates were entered as linear terms, consistent with the modelling strategy used in the main analyses.

Model results are reported as odds ratios (ORs) with 95% confidence intervals (CIs), obtained by exponentiation of regression coefficients. Statistical analyses were performed using R (glm function, *stats* package).

**Univariable linear mixed-effects models**

Associations between organ dysfunction phenotypes and total plasma piperacillin concentrations across repeated measurements were assessed using univariable linear mixed-effects models fitted to the full sampling dataset. Piperacillin concentration (mg/L) was modelled as a continuous outcome. A patient-specific random intercept was included to account for within-patient correlation arising from repeated sampling.

Organ dysfunction status was modelled as a four-level categorical variable, with no organ dysfunction as the reference category and isolated hepatic dysfunction, isolated renal dysfunction, and combined hepato-renal dysfunction as comparison groups. Classification of organ dysfunction was prespecified, with combined hepato-renal dysfunction prioritised over isolated hepatic or renal dysfunction to ensure mutually exclusive categories.

Additional univariable mixed-effects models were fitted for prespecified demographic, severity, comorbidity, and treatment-related covariates, including age, sex, body mass index, SAPS II, SOFA score, cirrhosis, chronic kidney disease, surgical admission, creatinine clearance, shock status, renal replacement therapy, invasive mechanical ventilation, serum albumin, total bilirubin, prothrombin rate, and daily piperacillin dose. Sex was coded as male versus female (reference).

For each predictor, analyses were performed on available observations for that variable. Models were fitted using maximum likelihood estimation to allow comparison across fixed-effect structures. Fixed-effect estimates are reported as regression coefficients (β) with 95% confidence intervals (Wald method), with associated p values obtained using the *lmerTest* package.

**Multivariable linear mixed-effects model**

A multivariable linear mixed-effects model was constructed to identify independent determinants of total plasma piperacillin concentrations across repeated measurements using the full sampling dataset. Piperacillin concentration (mg/L) was modelled as a continuous outcome, with a patient-specific random intercept included to account for within-patient correlation due to repeated sampling.

Fixed effects included variables associated with piperacillin concentrations at p < 0.20 in univariable mixed-effects analyses or considered clinically relevant namely age, sex, body mass index, SAPS II, SOFA score, organ dysfunction group (isolated hepatic, isolated renal, or combined hepato-renal dysfunction vs no dysfunction), shock status, total bilirubin concentration, and prothrombin rate. Variables reflecting overlapping aspects of organ dysfunction or renal support were not entered concurrently in the model to limit collinearity.

Observations with missing values were excluded from the multivariable analysis. Models were fitted using maximum likelihood estimation. Regression coefficients are reported as β estimates with 95% confidence intervals, representing the mean change in piperacillin concentration (mg/L) per unit increase in continuous predictors or relative to the reference category for categorical variables. Statistical inference was based on two-sided p values obtained using the *lmerTest* package.

**~~ANOVA analyses~~**

~~To further explore the relative contributions of daily maintenance dose, organ dysfunction, and their potential interaction to piperacillin exposure, a two-way analysis of variance (ANOVA) was performed. Log-transformed piperacillin concentration at the first therapeutic drug monitoring was modelled as the dependent variable. Daily maintenance dose (8, 12, or 16 g/day) and organ dysfunction group (no dysfunction, isolated hepatic dysfunction, isolated renal dysfunction, and combined hepato-renal dysfunction) were included as fixed categorical factors together with their interaction. Type II partial F-tests were used to assess the independent contribution of each factor. Partial eta-squared (partial η²) was calculated as a measure of effect size. The analysis was restricted to patients receiving one of the three principal maintenance dose regimens (n = 221).~~

**EXTENDED RESULTS**

**~~Two-way ANOVA~~**

~~Organ dysfunction group was independently associated with piperacillin concentration after adjustment for maintenance dose (F(3,215)=20.03, p<0.001; partial η²=0.22). In contrast, maintenance dose was not independently associated with piperacillin concentration (F(2,215)=2.14, p=0.120; partial η²=0.02). No significant interaction between maintenance dose and organ dysfunction group was observed (F(5,210)=0.82, p=0.537; partial η²=0.02), indicating that the relationship between administered maintenance dose and piperacillin concentration did not differ across organ dysfunction phenotypes (~~**~~Supplementary Table S7~~**~~).~~

**FIGURES AND TABLES**

**Supplementary Figure S1.** Piperacillin concentrations by target category, full set of samples

**Supplementary Figure S2.** ~~Total~~ Observed plasma piperacillin concentration by organ dysfunction group, full set of samples

**Supplementary Figure S3**. Distribution of piperacillin exposure categories according to daily maintenance dose and organ dysfunction group at first therapeutic drug monitoring.

Stacked bar plots showing the proportion of patients with underexposure (<80 mg/L), target attainment (80-160 mg/L), and overexposure (>160 mg/L) according to daily maintenance dose (8, 12, and 16 g/day) within each organ dysfunction phenotype. Analysis was restricted to the three principal maintenance dose regimens

**Supplementary Figure S4.** Longitudinal Transitions in Piperacillin Exposure Categories

**Supplementary Table S1.** The main demographic characteristics of patients, full set of samples

|  | **PIP concentration** | | | **Total** |
| --- | --- | --- | --- | --- |
|  | **Underdose** | **Target** | **Overdose** |  |
|  | n=240 (31%) | n=293 (37,8%) | n=242 (31,2%) | n=775 |
| *Characteristics on day of first Piperacillin assay* |  |  |  |  |
| **Creatinine clearance** (ml/min), median [IQR] | 143.4 [89.3–206.9] | 80.2 [48.7–138.1] | 28.4 [12.7–62.4] | 77.8 [32.0–152.7] |
| **Creatinine clearance** (ml/min), n (%) |  |  |  |  |
| > 120 | 137 (58%) | 80 (28%) | 13 (5%) | 230 (30%) |
| [60 – 120] | 61 (26%) | 83 (29%) | 40 (17%) | 184 (24%) |
| [30 – 59] | 18 (8%) | 44 (15%) | 39 (16%) | 101 (13%) |
| [10 – 29] | 10 (4%) | 34 (12%) | 56 (24%) | 100 (13%) |
| < 10 or RRT | 11 (5%) | 47 (16%) | 89 (38%) | 147 (19%) |
| **Albumin** (g/L), median [IQR] | 29.5 [26.0–33.0] | 29.0 [26.0–32.0] | 29.0 [26.0–33.0] | 29.0 [26.0–33.0] |
| **Bilirubin** (µmol/L), median [IQR] | 15.0 [8.8–34.0] | 25.0 [10.0–75.0] | 33.5 [13.0–136.8] | 23.0 [10.0–78.0] |
| **Prothrombin rate** (%), median [IQR] | 75.0 [63.0–87.0] | 73.0 [57.0–88.0] | 66.0 [49.2–82.0] | 72.0 [56.0–86.0] |
| *Piperacillin administration* |  |  |  |  |
| **Piperacillin dose per day** (mg/kg), median [IQR] | 183.9 [153.8–223.0] | 181.8 [140.4–225.4] | 183.9 [141.2–225.4] | 183.9 [144.6–225.4] |
| **Piperacillin dose per day** (g/day), median [IQR] | 16 [12–16] | 16 [12–16] | 12 [12–16] | 16 [12–16] |
| **Piperacillin concentration** (mg/L), mean (±SD) | 58.4 [46.6–70.3] | 113.3 [93.6–134.0] | 231.1 [188.1–350.0] | 113.6 [72.6–180.3] |

The daily piperacillin doses correspond to the dose being administered at each TDM time point (Day 1, Day 3, and Day 7).

**Supplementary Table S2.** Multinomial logistic regression — underexposure versus overexposure, full set of samples

|  | **Underdose vs Target** | | **Overdose vs Target** | | |
| --- | --- | --- | --- | --- | --- |
|  | OR [95% CI] | P value | OR [95% CI] | P value | |
| *Characteristics at inclusion* |  |  |  | |  |
| **Age** | 0.97 (0.96–0.99) | **0.000045** | 0.99 (0.98–1.01) | | 0.383 |
| **Gender male** | 1.65 (1.13–2.42) | **0.010** | 1.05 (0.73–1.50) | | 0.808 |
| **BMI** | 1.01 (0.99–1.03) | 0.530 | 0.98 (0.95–1.00) | | 0.070 |
| **Score** |  |  |  | |  |
| SAPS II | 0.99 (0.98–1.00) | **0.024** | 1.01 (1.00–1.02) | | **0.021** |
| SOFA | 0.90 (0.86–0.95) | **<0001** | 1.08 (1.04–1.13) | | **0.00027** |
| **Comorbid conditions** |  |  |  | |  |
| Cirrhosis | 0.76 (0.52–1.13) | 0.172 | 1.37 (0.95–1.98) | | 0.088 |
| Chronis kidney disease | 0.38 (0.21–0.70) | **0.0017** | 1.54 (1.00–2.39) | | 0.052 |
| **Surgical admission** | 1.37 (0.97–1.94) | 0.069 | 1.09 (0.77–1.54) | | 0.626 |
| **Liver dysfunction** | 0.67 (0.46–0.96) | **0.031** | 1.61 (1.14–2.27) | | **0.0072** |
| **Renal dysfunction** | 0.30 (0.20–0.45) | **<0.00001** | 3.74 (2.60–5.39) | | **<0.000001** |
| *Characteristics on day of first PIP assay* |  |  |  | |  |
| **Creatinine clearance** (ml/min) | 1.01 (1.00–1.01) | **<0.00001** | 0.98 (0.98–0.99) | | **<0.000001** |
| **Shock** | 0.51 (0.34–0.77) | **0.0016** | 0.98 (0.67–1.42) | | 0.914 |
| **Renal replacement therapy** | 0.22 (0.10–0.49) | **0.00018** | 1.57 (0.99–2.50) | | 0.057 |
| **Invasive ventilation** | 1.85 (1.30–2.64) | **0.00067** | 0.81 (0.57–1.13) | | 0.216 |
| **Albumin** | 1.02 (0.99–1.05) | 0.310 | 1.03 (1.00–1.06) | | **0.035** |
| **Bilirubin** (µmol/L) | 1.00 (0.99–1.00) | **0.0026** | 1.00 (1.00–1.00) | | **0.006** |
| **Prothrombin rate** | 1.01 (1.00–1.01) | 0.116 | 0.99 (0.98–1.00) | | **0.013** |
| *Piperacillin administration* |  |  |  | |  |
| **Piperacillin dose per day** | 1.08 (1.02–1.13) | **0.0055** | 0.97 (0.93–1.02) | | 0.314 |

**Supplementary Table S3a.** Multivariate model of underdose group versus target group, full set of samples

|  | **ß parameter** | **OR** | **95% CI** | **p-value** |
| --- | --- | --- | --- | --- |
| **Age** (per year) | −0.041 | 0.96 | 0.94 – 0.97 | **< 0.001** |
| **Gender male** | 0.89 | 2.44 | 1.56 – 3.86 | **< 0.001** |
| **Renal dysfunction** | −1.26 | 0.28 | 0.16 – 0.48 | **< 0.001** |
| **SOFA score** (per point) | −0.079 | 0.92 | 0.87 – 0.98 | **0.009** |
| **Shock** (Yes) | −0.76 | 0.47 | 0.29 – 0.74 | **0.002** |
| **Bilirubin (µmol/L, per unit)** | −0.003 | 1.00 | 0.99 – 1.00 | **0.027** |
| **SAPS II** (per point) | 0.015 | 1.01 | 1.00 – 1.03 | 0.060 |

**Supplementary Table S3b.** Multivariate model of overdose group versus target group, full set of samples

|  | **ß parameter** | **OR** | **95% CI** | **p-value** |
| --- | --- | --- | --- | --- |
| **Renal dysfunction** | 1.63 | 5.09 | 3.24 – 8.12 | **<0.001** |
| **Piperacillin dose** (g/day) | 0.094 | 1.10 | 1.03 – 1.17 | **0.003** |

**Supplementary Table S4.** Piperacillin Dosing and Exposure According to Organ Dysfunction Status at the first therapeutic drug monitoring measurement (Day 1, n = 350)

| **Group** | **Daily dose (g), median [IQR]** | **Underexposure** | **Target** | **Overexposure** |
| --- | --- | --- | --- | --- |
| **Augmented renal clearance** | 16 [16-16] | 48 (57%) | 30 (36%) | 6 (7%) |
| **No dysfunction** | 16 [16-16] | 24 (31%) | 35 (45%) | 19 (24%) |
| **Liver dysfunction** | 16 [16-16] | 8 (20%) | 19 (46%) | 14 (34%) |
| **Kidney dysfunction** | 12 [11-16] | 8 (9%) | 28 (32%) | 52 (59%) |
| **Liver & kidney dysfunction** | 12 [12-16] | 3 (5%) | 16 (27%) | 40 (68%) |

**Supplementary Table S5.** Univariable linear mixed-effects analyses of organ dysfunction and other covariates associated with total plasma piperacillin concentrations

|  | **ß parameter [95% CI]** | **P value** |
| --- | --- | --- |
| *Characteristics at inclusion* |  |  |
| **Age** | 0.77 [0.18; 1.36] | **0.011** |
| **Gender male** | -23.70 [-42.24; -5.16] | **0.012** |
| **BMI** | -1.43 [-2.63; -0.23] | **0.019** |
| **Score** |  |  |
| SAPS II | 1.07 [0.58; 1.56] | **<0.001** |
| SOFA | 5.33 [3.45; 7.22] | **<0.001** |
| **Comorbid conditions** |  |  |
| Cirrhosis | 13.14 [-6.15; 32.44] | 0.18 |
| Chronis kidney disease | 42.27 [18.43; 66.11] | **<0.001** |
| **Surgical admission** | -3.78 [-20.66; 13.11] | 0.660 |
| **Liver dysfunction** | 18.02 [-1.68; 37.73] | 0.073 |
| **Renal dysfunction** | 85.95 [67.55; 104.35] | **<0.001** |
| **Liver & Kidney dysfunction** | 109.87 [89.43; 130.31] | **<0.001** |
| *Characteristics on day of first PIP assay* |  |  |
| **Creatinine clearance** (ml/min) | -0.38 [-0.44; -0.31] | **<0.001** |
| **Shock** | 24.67 [5.76; 43.58] | **0.011** |
| **Renal replacement therapy** | 23.43 [0.25; 46.61] | **0.048** |
| **Invasive ventilation** | 4.58 [-10.10; 19.26] | 0.540 |
| **Albumin** | -0.16 [-1.44; 1.11] | 0.799 |
| **Bilirubin** (µmol/L) | 0.16 [0.09; 0.22] | **<0.001** |
| **Prothrombin rate** | -0.68 [-1.03; -0.33] | **<0.001** |
| *Piperacillin administration* |  |  |
| **Piperacillin dose per day** | -1.10 [-3.24; 1.05] | 0.317 |

Random intercept for patient: (1 | ID)

Organ dysfunction groups: no dysfunction as reference

**Supplementary Table S6.** Multivariable linear mixed-effects model assessing the independent association of organ dysfunction and other covariates with total plasma piperacillin concentrations

|  | **ß parameter [95% CI]** | **P value** |
| --- | --- | --- |
| **Age (per year)** | 0.79 [0.26; 1.31] | **0.003** |
| **Gender male** | −28.43 [−43.38; −13.49] | **<0.001** |
| **BMI** | −1.02 [−1.95; −0.09] | **0.032** |
| **Renal dysfunction** | 74.51 [55.83; 93.19] | **<0.001** |
| **Liver & Kidney dysfunction** | 80.11 [56.50; 103.72] | **<0.001** |
| **Bilirubin (µmol/L)** | 0.11 [0.04; 0.18] | **0.002** |
| **Prothrombin rate (%)** | −0.43 [−0.77; −0.09] | **0.013** |

**~~Supplementary Table S7.~~** ~~Two-way analysis of variance of log-transformed piperacillin concentrations at first therapeutic drug monitoring.~~

| **~~Factor~~** | **~~df~~** | **~~F~~** | **~~p-value~~** | **~~Partial η²~~** |
| --- | --- | --- | --- | --- |
| ~~Organ dysfunction group~~ | ~~3,215~~ | ~~20.03~~ | ~~<0.001~~ | ~~0.22~~ |
| ~~Daily maintenance dose~~ | ~~2,215~~ | ~~2.14~~ | ~~0.120~~ | ~~0.02~~ |
| ~~Dose × organ dysfunction interaction~~ | ~~5,210~~ | ~~0.82~~ | ~~0.537~~ |  |
